# From Traditional Risk Factors to Machine Learning Models: Advancing the Prediction of Anastomotic Leak and Other Major Complications in Colorectal Cancer Surgery

**DOI:** 10.3390/cancers18101668

**Published:** 2026-05-21

**Authors:** Sophia Tsokkou, Nikolaos Konstantinididis, Ioannis Konstantinidis, Menelaos Papakonstantinou, Filippos Alexandris, Despina Tokou, Konstantia Kotsani, Dimitrios Alexandrou, Dimitrios Giakoustidis, Alexandros Giakoustidis, Vasileios Papadopoulos, Petros Bangeas

**Affiliations:** 1 First Department of Surgery, General Hospital Papageorgiou, Aristotle University of Thessaloniki, 56429 Thessaloniki, Greece; stsokkou@auth.gr (S.T.); nikos4kanti@yahoo.gr (N.K.); menelaospap.md@gmail.com (M.P.); paterp39@gmail.com (F.A.); dtokou84@gmail.com (D.T.); konstantia.kotsani@gmail.com (K.K.); dimalexthess@gmail.com (D.A.); dgiak@auth.gr (D.G.); algiak@auth.gr (A.G.); papadvas@auth.gr (V.P.); 2 Laboratory of Histology-Embryology, Department of Medicine, Faculty of Health Sciences, Aristotle University of Thessaloniki, 54124 Thessaloniki, Greece; ikonsc@auth.gr

**Keywords:** colorectal cancer (CRC), artificial intelligence (AI), machine Learning (ML), deep learning (DL), anastomotic leak (AL)

## Abstract

Background: Colorectal cancer (CRC) represents a major global health burden, accounting for roughly 10% of all newly diagnosed cancers and cancer-related deaths worldwide. According to the World Health Organization, it is the third most diagnosed malignancy and the second leading cause of cancer mortality. Postoperative complications remain a significant concern after CRC resection, occurring in up to 50% of patients and contributing to increased morbidity, mortality, prolonged hospitalization, and substantial healthcare expenditure. Artificial intelligence (AI) has emerged as a transformative tool in modern healthcare, offering advanced capabilities in predictive analytics, clinical decision support, and personalized perioperative management. Methods: This review systematically evaluates the application of AI, specifically machine learning (ML) and deep learning (DL) algorithms, in the prediction of anastomotic leak (AL) and other major postoperative complications. In this context, AI models are generally used to refine risk stratification and enhance surgical decision-making. Results: A total of 13 studies were included, encompassing 15,105 patients. Across these studies, ML and DL algorithms consistently outperformed conventional statistical models in forecasting postoperative outcomes. Conclussions: Current evidence suggests that AI has substantial potential to improve perioperative risk prediction, support intraoperative decision-making, and personalize postoperative surveillance in patients undergoing CRC surgery. Methodological limitations, including a high risk of bias, limited external validation, heterogeneous outcome definitions, and inconsistent reporting, necessitate more robust, prospective, multicenter research before widespread clinical adoption can be realized.

## Simple Summary

Complications after colorectal cancer surgery, especially anastomotic leak, remain difficult to predict and can seriously affect recovery, survival, and quality of life. Traditional risk factors, such as age, frailty, nutrition, inflammation, tumor location, and operative difficulty, are useful but often cannot capture the complexity of postoperative risk. This review examines how artificial intelligence and machine learning models may improve the prediction of anastomotic leak and other major complications after colorectal cancer surgery. By summarizing the available studies, we show that these models can combine clinical, laboratory, imaging, and intraoperative data to identify high-risk patients more accurately than conventional approaches in many settings. Most current studies still have important limitations, including small datasets, inconsistent reporting, and limited external validation. Future high-quality research may help transform these tools into reliable decision-support systems that improve surgical planning, postoperative monitoring, and patient outcomes.

## 1. Introduction

Colorectal cancer (CRC) represents a significant global health concern, accounting for approximately 10% of all newly diagnosed cancers and cancer-related mortalities worldwide [1,2]. Surgical resection remains a basic curative strategy for CRC patients. Despite recent advancements in minimally invasive techniques, including laparoscopic, endoscopic, and robotic surgical options [3], as well as improvements in perioperative care, postoperative complications continue to present substantial clinical challenges [4]. These complications significantly and adversely affect patients, often resulting in prolonged hospital stays (HSs), increased healthcare costs and, in some cases, mortality [5].

Anastomotic leak (AL) is a critical and highly concerning complication in colorectal surgery, significantly affecting both short- and long-term patient outcomes. The reported incidence varies from 3% to 19%, contingent upon factors such as the anastomotic level, emergency status, and patient selection [6]. The repercussions of AL extend beyond immediate morbidity, as it increases the length of hospital stay, reoperation rates, and mortality. Additionally, it disrupts the timely initiation of adjuvant therapy, adversely affects oncologic survival in colorectal cancer patients, and often results in permanent stomas. AL is a major determinant of surgical quality and long-term oncologic success [5,6]. Despite ongoing advancements in perioperative care, surgical techniques, and enhanced recovery protocols, predicting which anastomoses will fail remains a formidable challenge. Numerous risk factors have been identified, reflecting the multifactorial nature of AL. Patient-related factors, such as malnutrition, smoking, diabetes, obesity, cardiopulmonary disease, and systemic inflammation, are recognized contributors. Disease-related factors include tumor location, neoadjuvant chemoradiotherapy, local sepsis, bowel obstruction, and anatomical constraints within the pelvis. Intraoperative factors, like poor tissue perfusion, high anastomotic tension, technical error, and emergency surgery, contribute to compromised healing [7]. The postoperative inflammatory response and infectious complications further modulate this risk. The pathophysiological background, as extensively described in the literature, involves a complex interplay of tissue ischemia, microbiome alterations, matrix degradation, and impaired collagen remodeling, ultimately leading to the failure of anastomotic integrity. Existing clinical scoring systems attempt to synthesize these variables into structured risk assessment tools; however, none have achieved widespread clinical adoption [8]. Their discriminative performance is modest, being limited by the complexity of AL, the heterogeneity of surgical populations, and the difficulty of capturing dynamic intraoperative factors. Moreover, these models typically rely on linear associations and cannot account for complex, non-linear interactions between variables. As a result, surgeons continue to rely on clinical judgment which, although irreplaceable, lacks standardized quantification or reproducibility across settings [6,7,8]. Over the past decade, artificial intelligence (AI) and machine learning (ML) have emerged as promising tools for risk stratification in complex clinical settings. In colorectal surgery, ML has already demonstrated utility in predicting morbidity, mortality, conversion to open surgery, and postoperative complications [9]. For AL specifically, AI models are particularly attractive given the large number of interacting risk factors and the incomplete understanding of the biological processes underlying anastomotic failure [10]. Early ML models incorporated demographic and perioperative variables, demonstrating improved predictive accuracy compared to conventional scoring systems. Subsequently, more sophisticated approaches have utilized deep neural networks, ensemble learning, and gradient boosting. Imaging-based models have also gained attention; radiomics, for example, allows for the extraction of quantitative imaging biomarkers from preoperative CT scans, capturing subtle textural or microarchitectural features not visible to the human eye [11]. Similarly, intraoperative near-infrared fluorescence imaging with indocyanine green (ICG) provides a platform for AI-assisted perfusion quantification, potentially offering a more reliable assessment of microvascular adequacy than the surgeon’s subjective visual evaluation [9,10,11]. Moreover, many studies lack external validation, which is essential for clinical application in real-world settings. Ethical considerations, data privacy, interpretability, and medico-legal implications also constitute significant barriers. While the potential of AI for the prediction of anastomotic leakage (AL) and other postoperative complications in colorectal surgery is considerable, its integration into routine clinical practice requires rigorous validation and standardized evaluation frameworks. To date, most AI-based models for AL prediction have been limited by heterogeneity in study design, small and often single-center training cohorts, and a lack of external validation factors that significantly constrain their clinical applicability. Moreover, no existing review has comprehensively bridged established surgical risk determinants with emerging computational methodologies. Addressing this gap is critical for translating AI from theoretical promise into practical, evidence-based intraoperative decision support. The aim of our review is to evaluate the current role played by AI and machine learning models in predicting complications after colorectal surgery by explicitly linking traditional clinical risk factors, underlying pathophysiological mechanisms, and modern computational approaches. By integrating insights from conventional surgical knowledge with advances in data-driven modeling, this review synthesizes the strengths and limitations of existing AI-based prediction tools, highlights the methodological challenges that impede widespread implementation, and outlines key directions for future development. Ultimately, we explore how these technologies may evolve to support real-time surgical decision-making and improve patient outcomes.

### 1.1. Risk Factors Contributing to Postoperative Complications

**Figure 1:**
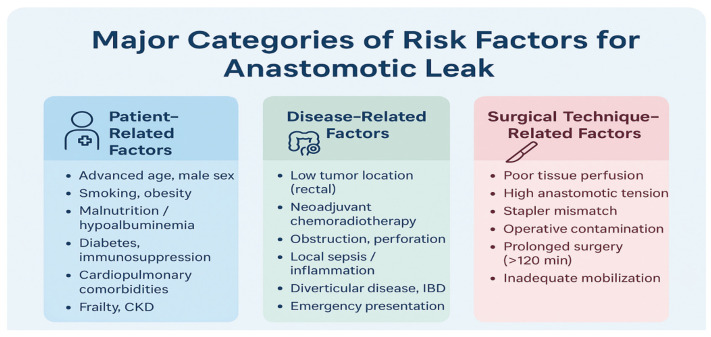
Risk factors for anastomotic leak after colorectal surgery.

Postoperative complications following colorectal surgery emerge from a complex interplay of patient-related, disease-related, and perioperative factors (Figure 1). Advanced age, frailty, and the presence of comorbidities—particularly neurological and cardiorespiratory conditions—consistently increase postoperative vulnerability.

**Figure 2:**
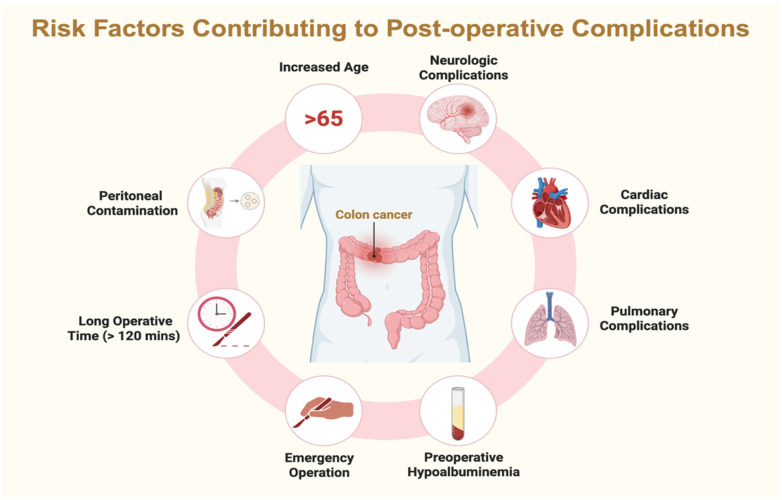
Risk factors for postoperative complications.

Preoperative hypoalbuminemia, indicative of both systemic inflammation and malnutrition, is a well-recognized predictor of infectious and non-infectious complications. Operative characteristics also play a significant role; emergency surgery, prolonged operative duration beyond 120 min, and peritoneal contamination substantially elevate the risk of adverse postoperative outcomes (Figure 2).

Evidence from extensive multicenter cohorts underscores this multifactorial nature. In a prospective study conducted across 81 French hospitals involving 1421 patients treated for colorectal cancer or diverticular disease, several variables were identified as independent predictors of postoperative mortality. Emergency surgery, preoperative weight loss exceeding 10%, neurological comorbidities, and age over 70 years cumulatively contributed to heightened risk. Overall postoperative morbidity reached 35%, with wound infection, anastomotic leak, hemorrhage, stoma-related complications, prolonged ileus, and cardiorespiratory events emerging as the most common complications. Hospital stays were significantly extended, averaging 17 days, particularly among patients undergoing emergency procedures, those with underlying malignancy, and those treated in public hospitals [12].

In additional studies focusing on elderly surgical populations, sarcopenia—assessed via diminished psoas muscle area on preoperative CT—emerged as a strong independent predictor of major complications; along with hypoalbuminemia, it consistently signaled physiological fragility and correlated closely with prolonged hospital stay. Although 30-day readmission rates were low (4%) and 90-day mortality modest (1%), reduced psoas density reliably identified patients at increased risk of postoperative deterioration [13,14]. Taken together, these findings highlight the importance of comprehensive preoperative evaluation, integrating frailty indices and CT-derived markers of sarcopenia to identify high-risk individuals who may benefit from more tailored perioperative strategies.

The role of the intestinal microbiome in anastomotic healing adds further biological complexity. Microbial dysbiosis, particularly the overrepresentation of collagenase-producing species such as *Enterococcus faecalis* and *Pseudomonas aeruginosa*, has been implicated in collagen degradation at the anastomotic site. Altered biofilm behavior, shaped by bowel preparation or exposure to broad-spectrum antibiotics, may further promote bacterial phenotypes that weaken the extracellular matrix and impair mucosal regeneration. These microbial influences interact intimately with host immune and inflammatory responses; for example, elevated postoperative inflammatory markers, including CRP and aberrant neutrophil-to-lymphocyte ratios, have been associated with an increased likelihood of clinically significant anastomotic leak, reflecting this complex microbe–immune interface [15]. Collectively, these clinical, physiological, and biological dimensions illustrate postoperative morbidity as a deeply multifaceted phenomenon. In the specific context of anastomotic healing, the convergence of mechanical stressors, host vulnerability, microbial influences, and intraoperative technical considerations underscores the need for multidimensional risk assessment frameworks. Such integrated models, combining patient physiology, disease characteristics, operative variables, and emerging insights from microbiome science, represent the most promising pathway toward more accurate prediction of anastomotic failure and, ultimately, improved surgical outcomes.

### 1.2. Postoperative Complications Following CRC Resection

**Figure 3:**
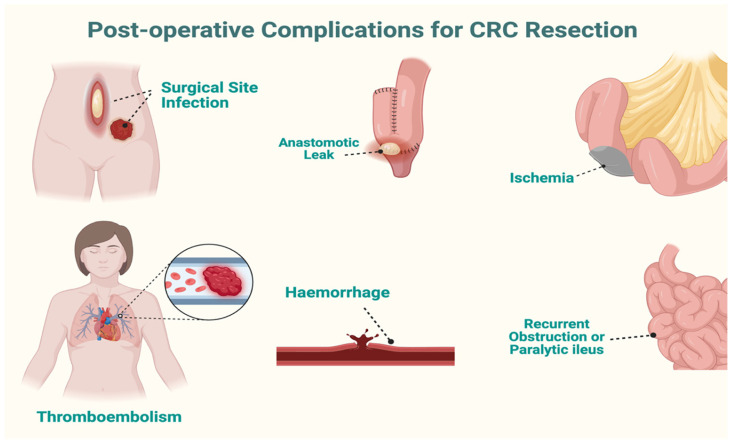
Postoperative complications.

Postoperative complications following colorectal cancer (CRC) resection affect up to 50% of patients and markedly increase morbidity, mortality, hospital stay, and healthcare costs [16]. Approximately 40% of individuals experience at least one complication, ranging from surgical events such as anastomotic leak (AL), hemorrhage, and wound infection to systemic complications including pneumonia, thromboembolism, and sepsis (Figure 3) [17].

The Clavien–Dindo classification provides a standardized and widely adopted framework for grading severity, and higher-grade complications are strongly associated with delayed recovery and impaired long-term oncological outcomes [17]. Notably, complications often interrupt or postpone adjuvant chemotherapy, a critical determinant of survival in stage III CRC. In a large retrospective study, patients with postoperative complications exhibited significantly lower 5-year overall and disease-free survival compared to those without complications [18,19]. Among postoperative complications, AL is particularly concerning, with incidence rates ranging from 2% to 19% depending on tumor location, surgical technique, and patient characteristics; in distal rectal cancer, rates may reach 24% [20]. AL carries substantial morbidity, increased mortality risk, a higher likelihood of permanent stoma, and adverse oncological consequences including local recurrence and reduced long-term survival [18,19].

Risk factors for AL are multifactorial and span **patient-related, disease-related, and technical domains**. Patient-related factors include male sex, obesity, diabetes, chronic pulmonary disease, smoking, alcohol use, and advanced age, all of which impair tissue healing or increase technical complexity [20,21]. Disease-related contributors include tumor location, neoadjuvant chemoradiotherapy, obstruction, and local sepsis. Technical and intraoperative factors such as prolonged operative time (>200 min), blood loss > 200 mL, intraoperative transfusions, and low anastomotic height (≤5–7 cm from the anal verge) further elevate risk by reducing perfusion and increasing procedural difficulty [20]. Postoperative factors, including anemia, corticosteroid or NSAID use, and perioperative transfusions, may also compromise anastomotic healing. Although diverting stomas do not reduce the incidence of AL, they mitigate its clinical consequences by lowering the need for reoperation and limiting sepsis-related morbidity [20]. A detailed understanding of these interrelated risk factors forms the clinical foundation upon which ML-based models aim to enhance predictive accuracy and guide perioperative decision-making.

### 1.3. Artificial Intelligence in Surgical Prognosis

Artificial intelligence (AI) has rapidly evolved into a transformative force in modern healthcare, offering unprecedented capabilities in predictive analytics, clinical decision-making, and outcome optimization. Particularly through machine learning (ML) and deep learning (DL) algorithms, AI in colorectal surgery has shown considerable promise in terms of enhancing prognostic accuracy beyond the limitations of traditional statistical approaches [21]. Conventional models, which rely on linear assumptions and struggle to integrate complex, interdependent variables, are inherently constrained when applied to the multifactorial landscape of surgical complications. In contrast, ML techniques can process high-dimensional, multimodal datasets and uncover subtle, non-linear patterns that would otherwise remain undetected. AI-driven models have already been applied across a broad spectrum of clinical scenarios, including prediction of postoperative morbidity, mortality, conversion to open surgery, surgical site infections, prolonged ileus and, most critically, anastomotic leak (AL). By synthesizing preoperative characteristics, intraoperative metrics, and early postoperative indicators, ML systems can generate dynamic, patient-specific risk estimates, enabling real-time support for intraoperative and perioperative decision-making [22,23,24]. Beyond risk prediction, AI holds transformative potential in shaping perioperative management, especially in terms of identifying high-risk patients, guiding tailored operative strategies, and informing targeted postoperative surveillance. Early evidence suggests that AI-enhanced risk stratification may enable clinicians to intervene earlier through diversion, intensified monitoring, or the optimization of modifiable risk factors, thereby improving clinical outcomes and streamlining resource utilization across surgical pathways [24,25,26,27,28]. As these technologies progress, their effective integration into routine surgical practice will depend on rigorous external validation, model interpretability, and seamless incorporation into established clinical workflows.

### 1.4. Objective

The aim of this review is to provide a comprehensive and critical assessment of artificial intelligence applications—specifically, machine learning and deep learning methodologies—for the prediction of anastomotic leak and other major postoperative complications following colorectal cancer resection. By synthesizing evidence across the entire perioperative continuum, this review explores how AI-based models may enhance traditional risk assessment, offer real-time decision support, and ultimately improve clinical outcomes through the earlier and more accurate identification of high-risk patients.

## 2. Methodology

### 2.1. Study Framework (PICO/PICOS)

This systematic review was conducted utilizing a PICOS framework specifically adapted for studies that develop or validate artificial intelligence-based predictive models in colorectal cancer (CRC) surgery. The **Population (P)** consisted of published clinical studies involving adult patients undergoing colorectal resection in either colonic or rectal surgery via open, laparoscopic, robotic, or hybrid approaches, wherein machine learning or deep learning techniques were employed for postoperative risk prediction. The **Intervention/Index** Test **(I)** encompassed any artificial intelligence-driven predictive methodology, including Random Forest, XGBoost, LightGBM, Support Vector Machines, neural networks, GRU-D architectures, BI-LSTM models, automated AI platforms, or hybrid approaches integrating intraoperative imaging or biochemical data. The **Comparator (C)** included conventional statistical models (e.g., logistic regression, ACS-NSQIP calculators, and the Colon Leakage Score) when reported; however, studies lacking explicit comparators were also considered eligible, recognizing that many AI models remain exploratory and in the early stages of development. The **Outcomes (O)** included all postoperative complications predicted by the included models. Anastomotic leak (AL) was the most frequently examined endpoint, but secondary outcomes such as surgical site and organ-space infections, prolonged ileus, cardiopulmonary events, postoperative bleeding, readmission, length of stay, mortality, and functional outcomes, including low anterior resection syndrome (LARS), were also evaluated across the literature. The **Study Design (S)** comprised retrospective and prospective cohort studies (either single-center or multicenter) that developed, internally validated, or externally validated AI-based prediction models. Exclusion criteria included randomized trials (none identified), editorials, commentaries, narrative reviews, conference abstracts without full manuscripts, non-English publications, and studies lacking quantitative model performance metrics.

### 2.2. Search Strategy and Study Selection

A systematic literature search was conducted in accordance with the **PRISMA 2020** guidelines to identify studies evaluating AI-based prediction of postoperative complications after CRC resection. Searches were performed across **PubMed (MEDLINE), Cochrane Library, Embase, Scopus, and ScienceDirect**, without date restrictions, using the Boolean query **(“Artificial Intelligence”) AND (“Predictive Modeling”) AND (“Postoperative Complications”) AND (“Colorectal Cancer”)**.

Eligible studies were required to:

1. Evaluate postoperative outcomes following CRC surgery;
2. Apply an ML or DL model for prediction;
3. Report performance metrics (e.g., AUROC, accuracy, sensitivity, or specificity);
4. Provide full-text original research.

**Figure 4:**
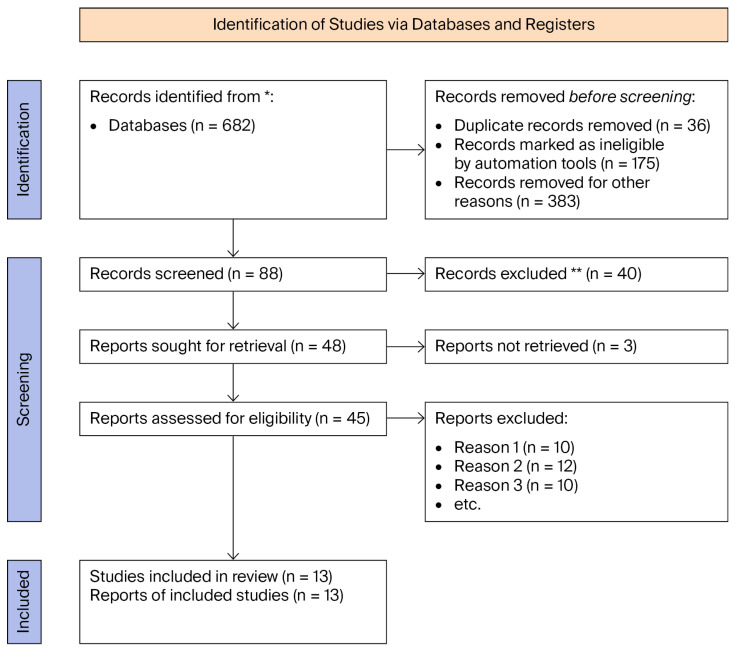
PRISMA chart. * Database included PubMed, Scopus and Web of Science. ** Records Excluded after title and abstract screening based on predefined eligibility Criteria.

Exclusion criteria were **R1** for non-English language, **R2** for studies unrelated to CRC surgery, and **R3** for abstracts without a full text, editorials, commentaries, narrative reviews, or studies lacking quantitative model performance assessment. Screening was performed in the following two stages (Figure 4): Title/abstract screening was followed by full-text evaluation according to predefined criteria. Reference lists of included studies were also screened to identify additional eligible articles.

## 3. Results

A total of 13 studies satisfied the inclusion criteria, representing a combined cohort of 15,105 patients (Table 1). The included studies comprised both multicenter and single-center cohort designs, employing retrospective and prospective methodologies; notably, no randomized controlled trials (RCTs) were identified. Publication years ranged from 2017 to 2025, with most studies appearing after 2020, reflecting the rapid acceleration and maturation of AI applications in colorectal surgery during the current decade.

**Figure 5:**
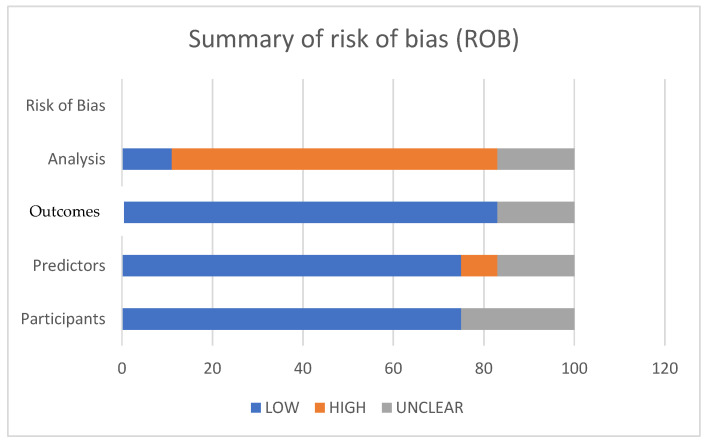
Risk of bias.

**Figure 6:**
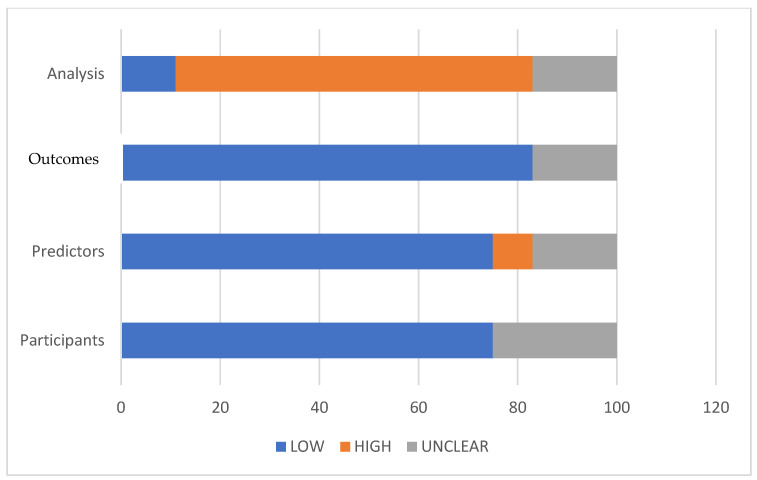
Applicability.

The identification, screening, eligibility assessment, and final inclusion stages are summarized in the PRISMA 2020 flow diagram for this review (Figure 4). A PROBAST-based quality assessment of the 13 included studies identified significant methodological variability, with most investigations exhibiting a high or unclear risk of bias. Only a minority of studies demonstrated a low risk across multiple PROBAST domains, and none met all the criteria (Figure 5 and Figure 6). Additionally, the AUC (area under the ROC) was used as the primary metric for model discrimination, reflecting each algorithm’s ability to distinguish between patients who developed postoperative complications—particularly anastomotic leak (AL)—and those who did not.

### 3.1. Targeted Subset Synthesis for Anastomotic Leak Prediction

**Figure 7:**
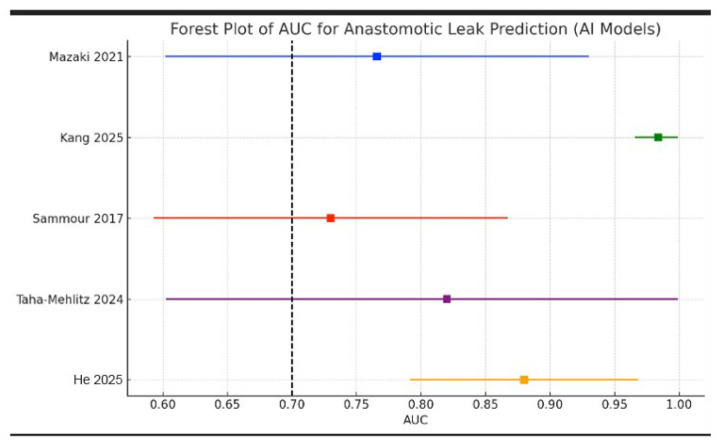
AUROC rates for AL studies [25,31,32,34,37].

Since AL represents the most clinically important postoperative event, we performed a targeted subset synthesis of studies that reported discrimination performance (AUROC) for AL prediction, as summarized below (Figure 7). Due to heterogeneity in study design, predictors, outcome definition, and validation strategies, a quantitative meta-analysis was not pursued; instead, our approach enabled a direct comparison across models using a common metric.

Targeted subset synthesis included five studies (He 2025, Kang 2025, Mazaki 2021, Sammour 2017, Tahamehlitz 2024) [25,31,32,34,37]. As shown in Figure 7, the reported AUROC values in smaller retrospective cohorts ranged from **0.766** (Mazaki) to **0.820** (Tahamehlitz). In the largest multicenter study, discrimination in internal validation was excellent (**AUROC 0.984**). Sammour et al. reported a broader discriminatory performance range (**0.73–0.96**), depicted as a midpoint with error bars. He et al. incorporated intraoperative perfusion information and reported a strong signal associated with AL risk using a non-AUROC metric; thus, this study is described narratively rather than included in the AUROC comparison.

### 3.2. Study-by-Study Synthesis Across Postoperative Outcomes

Mazaki et al. evaluated 256 left-sided CRC resections using an AutoAI platform (Prediction One), achieving an AUC of 0.766 for AL prediction and demonstrating lower leak rates with triple-row staplers. Masum et al. analyzed 4336 patients from a UK NHS trust, showing that BI-LSTM and SVR models predicted length of stay, readmission, and 31-day mortality with accuracies of 83%, 87.5%, and 96.6%, respectively, emphasizing the advantage of incorporating comprehensive variable sets. Chen et al. assessed 1067 patients undergoing simultaneous colorectal and liver resections for CRLM and reported that Random Forest models predicted postoperative complications (AUC 0.716) and long-term oncologic outcomes, with KRAS, BRAF, and MMR status emerging as key variables. Tian et al. evaluated 512 elderly CRC patients ≥ 65 years using the MGA-XGBoost algorithm, and achieved an AUC of 0.862 for infectious complications, highlighting inflammatory and nutritional indices, particularly the lymphocyte-to-C-reactive protein ratio (LCR), as dominant predictors. He et al. introduced an intraoperative perfusion assessment model using ICG fluorescence imaging and XGBoost in 68 rectal cancer patients, achieving an R^2^ of 97.2% for perfusion quantification and demonstrating strong associations with postoperative complications. Anania et al. analyzed 2013 registry patients and found that deep learning models predicted overall postoperative morbidity with an accuracy of 0.86, though AL-specific prediction was not examined. Wang et al. studied 493 rectal cancer patients and reported that Random Forest achieved the highest performance for early postoperative complications (AUC 0.880), with inflammatory markers such as PNI and IPI identified as major predictors. Sammour et al. validated the anastomoticleak.com calculator in 402 colon cancer patients, demonstrating superior discrimination compared with ACS-NSQIP and the Colon Leakage Score (AUC 0.73 overall; 0.96 for left colectomy). Fang et al. evaluated 109 elderly patients undergoing robotic NOSE surgery and found that XGBoost achieved an AUC of 0.92, with age, coronary heart disease, ASA grade, and hemoglobin emerging as key predictors. Ruan et al. analyzed 3534 CRC patients using time series models and demonstrated that GRU-D architectures outperformed logistic regression in the prediction of wound and organ-space infections (AUC 0.80 vs. 0.69), highlighting the value of dynamic physiological data. Huang et al. applied multiple ML models to 342 patients to predict LARS, with Random Forest achieving an AUC of 0.858 and tumor-related anatomical variables dominating feature importance. Kang et al. conducted one of the largest AL-focused investigations (1818 patients), demonstrating the excellent internal performance of an XGBoost model (AUC 0.984) but substantial degradation upon external validation (AUC 0.703); serum calcium was the strongest predictor. The Swiss pilot study by Taha-Mehlitz et al. applied logistic regression, Random Forest, and gradient boosting to preoperative biochemical and radiologic variables, demonstrating encouraging internal discrimination but limited generalizability due to the small sample size and lack of external validation.

## 4. Discussion

Artificial intelligence is increasingly transforming postoperative risk prediction in CRC surgery, with growing evidence indicating that machine learning (ML) and deep learning (DL) approaches can achieve clinically meaningful discrimination across a spectrum of postoperative outcomes, including anastomotic leakage (AL), infectious morbidity, prolonged ileus, cardiopulmonary events, readmission, and functional impairment. By integrating multidimensional perioperative data, including biochemical and inflammatory markers, intraoperative perfusion metrics, genomic profiles, and dynamic time series variables, AI facilitates granular risk stratification, supports intraoperative decision-making, and enables increasingly personalized perioperative care.

Across the 13 studies included in this review, several families of AI models recurred (e.g., XGBoost, Random Forest, automated AI platforms, and recurrent neural networks such as BI-LSTM and GRU-D), reflecting a broad methodological landscape. A key strength of AI in this context is its ability to model complex, non-linear relationships among predictors, which may be challenging for conventional regression-based approaches. Both established and emerging predictors were repeatedly highlighted, including age, ASA grade, comorbidity burden, operative duration, surgical approach, and stoma formation. Inflammatory and nutritional indices, such as NLR, PLR, LMR, PNI, and particularly the lymphocyte–C-reactive protein ratio (LCR), frequently ranked among the influential variables, especially in older cohorts. Novel physiological features were also introduced; notably, He et al. combined ICG fluorescence angiography with ML-based perfusion assessment and reported strong associations between perfusion deficits and postoperative complications, supporting the potential role of intraoperative physiological signals in AL-related risk assessment [29,31,35].

Several investigations suggested actionable clinical utility beyond performance metrics; for example, Mazaki et al. reported that an AutoAI approach could inform technical decisions such as stapler selection [22], while Chen et al. proposed individualized prediction tools in the setting of simultaneous colorectal and liver resections [29]. In elderly populations, where baseline vulnerability is higher and conventional calculators may underperform [29,30,35,36,38], focusing on patients ≥ 65 years resulted in encouraging discrimination for infectious and cardiopulmonary complications and AL-related morbidity, suggesting that AI-based stratification could be particularly valuable in high-risk subgroups.

These findings indicate that AI may be especially valuable in populations in which traditional risk calculators underperform. Methodological inconsistencies were prevalent in these studies and the definitions of AL and postoperative infections varied significantly, complicating cross-study comparisons; for example, essential steps in model development, such as feature preprocessing, hyperparameter optimization, calibration assessment, and the handling of missing data, were reported inconsistently. These weaknesses were evident in the PROBAST evaluation, where most studies were classified as having a high overall risk of bias, despite low or unclear ratings in several individual domains. Notably, the PROBAST tool does not calculate overall risk as an average; rather, a single high-risk domain automatically assigns a high-risk label to the entire study (Figure 5 and Figure 6). In the present review, the Analysis domain was the primary contributor to the high overall risk, due to the insufficient handling of missing data, lack of calibration, inadequate assessment of overfitting, and limited external validation. This structural characteristic accounts for the disproportionately elevated overall bias ratings observed in the included studies. Since the early recognition of postoperative complications remains a critical determinant of patient outcomes, AI-enhanced models have direct surgical relevance beyond their predictive accuracy. Anastomotic leak, infectious morbidity, and cardiopulmonary deterioration often follow a rapid and clinically deceptive course, and delayed diagnosis significantly increases sepsis rates, reintervention requirements, prolonged hospitalization, and mortality. Timely identification of high-risk patients enables closer monitoring, personalized perioperative pathways, and earlier therapeutic interventions, including expedited imaging, targeted antimicrobial therapy, and prompt surgical or endoscopic management. From a surgical standpoint, tools capable of detecting subtle physiological deviations or radiological patterns prior to overt clinical deterioration offer the potential to shift management from reactive to anticipatory, thereby reducing both morbidity and mortality. In this context, AI-supported early warning systems could meaningfully complement clinical judgment, especially in high-volume units where postoperative trajectories vary widely between patients. Several limitations impede the immediate integration of artificial intelligence (AI) into routine surgical practices. External validation is rare, and models frequently exhibit substantial performance decline when applied outside their development cohorts; for instance, Kang et al. reported an internal area under the receiver operating characteristic (AUROC) of 0.984, but this decreased to 0.703 during external testing [34]. Similarly, the Swiss pilot study by Taha-Mehlitz et al. showed promising internal discrimination but lacked broader validation, thereby limiting its generalizability [39,40,41]. The predominance of retrospective, single-center studies further exacerbates concerns regarding overfitting, selection bias, and limited applicability across diverse clinical settings. Despite these limitations, the trajectory of AI integration into colorectal cancer (CRC) surgery is overwhelmingly positive. Evidence increasingly suggests a shift from experimental modeling toward clinically meaningful decision support. As methodological rigor improves, datasets expand, and interpretability tools such as SHAP become more prevalent, AI-based predictive models are likely to play an increasingly crucial role in surgical planning, intraoperative assessment, and postoperative surveillance [39]. Future research should prioritize the development of large, multicenter prospective datasets with harmonized outcome definitions and standardized reporting frameworks for prediction-model development. Alongside rigorous external validation with performance stability and decision curve analyses to evaluate clinical utility, the use of SHAP-based interpretability is essential for building clinical trust [40]. Additionally, the integration of AI tools into user-friendly clinical workflows is crucial. Although only a minority of the included studies incorporated imaging-derived features, radiomics represents a promising frontier in AI-assisted risk prediction for colorectal surgery. Radiomics facilitates the extraction of high-dimensional quantitative biomarkers from routine preoperative CT scans, capturing the subtle textural, geometric, and microarchitectural characteristics of bowel wall integrity, mesenteric vascularity, and tumor biology that are imperceptible in human evaluation. Preliminary research in colorectal surgery has demonstrated that radiomic signatures derived from perianastomotic tissue, mesorectal fat, or the tumor microenvironment may predict impaired healing, infectious complications, or the propensity for anastomotic failure [39,41]. Radiogenomic correlations linking CT-based texture features with KRAS, BRAF, or MMR status further underscore the potential of integrating genomic and imaging biomarkers within unified machine learning frameworks [40]. When combined with intraoperative perfusion metrics obtained through ICG fluorescence, radiomics may enable the construction of multimodal models that assess both structural and physiological determinants of anastomotic integrity [39,40]. Although such imaging-based approaches remain underrepresented in the current literature and were not captured in most of the included studies, they constitute a critical future research direction with substantial potential to enhance patient-specific risk stratification. Overall, AI has the potential to transform perioperative care in CRC surgery, but it’s safe and effective implementation requires rigorous validation, methodological transparency, and careful clinical integration [39,40,41,42].

Despite these promising advances, the existing literature is constrained by notable methodological limitations. Most studies are retrospective, single-center, and exhibit a high or unclear risk of bias (Figure 5 and Figure 6), particularly concerning participant selection, outcome definitions, missing-data handling, and analytical transparency. External validation remains uncommon, and calibration reporting is inconsistent, raising concerns about reproducibility, overfitting, and real-world generalizability. These limitations underscore the need for well-designed multicenter prospective datasets, standardized reporting frameworks, transparent model development pipelines, and robust external validation procedures. Nevertheless, the overall trajectory of AI integration into CRC surgery is distinctly positive. As methodological rigor improves and as intuitive, clinician-friendly interfaces continue to emerge, AI-enhanced prediction tools are poised to become integral components of surgical planning, intraoperative assessment, and postoperative surveillance. With continued refinement and responsible implementation, artificial intelligence has the potential to meaningfully improve surgical outcomes, enhance patient safety, and optimize resource utilization across the entire continuum of colorectal cancer care.

## 5. Conclusions

Artificial intelligence is increasingly transforming postoperative risk prediction in colorectal surgery, with machine and deep learning models consistently exhibiting superior discriminative capacity compared to traditional statistical methods. In the 13 studies included in this systematic review, AI-based tools demonstrated strong performance in predicting a wide range of postoperative complications, highlighting the growing maturity and clinical relevance of computational modeling in colorectal surgery. Among the outcomes examined, anastomotic leak (AL) emerged as the most clinically significant complication, with AI showing some of the most compelling results in this area. Although the current literature remains methodologically heterogeneous, a focused subgroup synthesis of five AL-specific models revealed consistently high discriminative performance (AUC 0.73–0.984). This targeted analysis, supported by a comparative logit-transformed plot, illustrates the clear potential of AI-driven approaches to enhance the early recognition of AL, an outcome with profound implications for morbidity, mortality, reintervention, and long-term oncologic survival. Rather than representing a limitation of this review, the inclusion of studies covering a broader range of complications provides a comprehensive understanding of how AI functions throughout the entire perioperative continuum. This broader perspective underscores an important observation: models that perform well for AL tend to perform well across other major postoperative endpoints, suggesting that AL prediction may serve as a benchmark for evaluating the robustness of AI-based perioperative tools. Looking forward, the field is expected to benefit from harmonized definitions, rigorous external validation, and integration of emerging modalities, such as radiomics and intraoperative perfusion analytics. With continued methodological refinement and responsible clinical integration, artificial intelligence has the potential to become a pivotal adjunct to surgical judgment, enabling earlier intervention, reducing postoperative morbidity, and improving outcomes for patients undergoing colorectal cancer resection.

## Figures and Tables

**Table 1:** Included studies.

Author	Year	Study Design	Sample Size	AI Methods	Outcomes	Key Findings	Clinical Conclusion
Anania [29]	2025	Multicenter Retrospective	2013	DLMN	Overall Complications	Accuracy 0.86	Better periop Risk
Fang [30]	2025	Retrospective	109	XGB, LR + SHAP	AL, SSI	AUC 0.92	Strong elderly prediction
He [31]	2025	Prospective Retrospective	68	XGBoost + perfusion model	AL	R^2^ = 97%; low perfusion → AL	Improves intraop decisions
Kang [32]	2025	Multicenter retrospective	1818	XGB + SHAP	AL	AUC 0.984 int/0.703 ext	Calcium biomarker
Chen [27]	2024	Multicenter Retrospective	1070	Random Forrest	ComplicationsPFS/OS	KRAS/BRAF/MMR key	Guides CRLM therapy
Huang [33]	2024	Retrospective Cohort	342	LR, SVM, RF	LARS	RF AUC 0.858	Predicts functional outcomes
Tahamehlitz [34]	2024	Retrospective	152	RF, XGB, LR	AL	AUC 0.82	Swiss real-time AL monitoring
Tian [28]	2024	Retrospective	512	MGA-XGB, XGB, LGBM	Infectious complications	AUC 0.862; LCR strong	Improves elderly stratification
Wang [35]	2023	Retrospective	493	RF, SVM, LR	AL, ileus, SSI	RF AUC 0.88	Biomarker + ML improves prediction
Masum [26]	2022	Retrospective	4336	SVR, BI-LSTM, RF	LOS, readmission, mortality	Accuracy up to 96.6%	Supports resource planning
Ruan [36]	2022	Retrospective	3534	GRU-D	Infections	AUROC 0.80	Dynamic real-time PSC
Mazaki [25]	2021	Retrospective	256	Auto-AI (NN + GBM)	AL	AI AUC 0.766; triple-row stapler ↓ AL	AI informs stapler selection
Sammour [37]	2017	Retrospective	402	IBM Watson	AL	UROC 0.73–0.96	I > NSQIP/CLS

## Data Availability

No new data were created or analyzed in this study. This review is based on previously published articles, which are available from the respective publishers. Data sharing is not applicable to this article.
